# Spatio-temporal parameters for optical probing of neuronal activity

**DOI:** 10.1007/s12551-021-00780-2

**Published:** 2021-02-23

**Authors:** Vincent R. Daria, Michael Lawrence Castañares, Hans-A. Bachor

**Affiliations:** 1 Research School of Physics, The Australian National University, Canberra, Australia; 2 John Curtin School of Medical Research, The Australian National University, Canberra, Australia

**Keywords:** Neuronal circuits, Neuronal activity, Calcium Imaging, Voltage Imaging, Microscopy, Multi-photon microscopy

## Abstract

The challenge to understand the complex neuronal circuit functions in the mammalian brain has brought about a revolution in light-based neurotechnologies and optogenetic tools. However, while recent seminal works have shown excellent insights on the processing of basic functions such as sensory perception, memory, and navigation, understanding more complex brain functions is still unattainable with current technologies. We are just scratching the surface, both literally and figuratively. Yet, the path towards fully understanding the brain is not totally uncertain. Recent rapid technological advancements have allowed us to analyze the processing of signals within dendritic arborizations of single neurons and within neuronal circuits. Understanding the circuit dynamics in the brain requires a good appreciation of the spatial and temporal properties of neuronal activity. Here, we assess the spatio-temporal parameters of neuronal responses and match them with suitable light-based neurotechnologies as well as photochemical and optogenetic tools. We focus on the spatial range that includes dendrites and certain brain regions (e.g., cortex and hippocampus) that constitute neuronal circuits. We also review some temporal characteristics of some proteins and ion channels responsible for certain neuronal functions. With the aid of the photochemical and optogenetic markers, we can use light to visualize the circuit dynamics of a functioning brain. The challenge to understand how the brain works continue to excite scientists as research questions begin to link macroscopic and microscopic units of brain circuits.

## Introduction

Understanding the detailed computing operations in the brain is key to the advancement of analyzing and treating neurological illnesses. This venture requires mapping of the processes in the complex network of neurons and the function of individual neurons with high spatial and temporal resolution. Ideally, with data obtained from the ionic activity within a neuron, and across networks of neurons, the processing of information in brain circuits can be decoded. In the last decade, optical technologies have systematically improved and this has led to rapid developments of new methods to analyze the computing operations within dendrites of single neurons and neuronal populations (Daria and Bachor 2015; Ji et al. 2016; Yang and Yuste 2017; Go and Daria 2017). Analyzing how neurons process information requires time-resolved data obtained from multiple locations in three dimensions (3D). Within a single-neuron, multiple synapses are received along its dendritic tree, processed and transmitted to its neighboring neurons. When viewed from a larger scale involving neuronal circuits, the timing of signaling between spatially separated neurons constitutes complex circuit functions that are correlated with sensory input or behavior. Hence, the ability to probe these spatio-temporal events occurring in single neurons and neuronal circuits is vital to decode how information is processed in our brain.

The spatio-temporal dynamics of the computing operations in the brain can be analyzed from the sub-microscopic scale involving molecules/proteins to macroscopic scale comprising brain regions that constitute neuronal circuits. The membrane of a neuron holds several proteins and ion-channels that gate ions in and out of the neuron (Hille 1986). The temporal properties of ion channels as well as their spatial distribution across different dendritic regions are fundamental attributes that influence the neuron’s function. When a neuron receives synaptic inputs, ion channels gate positive ions and consequently depolarizing the cell. Multiple synaptic inputs along different dendritic regions build-up to reach a threshold for firing an output or an action potential (AP). Electrophysiological recordings can analyze the temporal dynamics of membrane depolarizations, such as an excitatory post-synaptic potential (EPSP) that occurs when a neuron receives a synaptic input (Sakmann and Neher 1984). Multiple electrodes have been used to probe different locations of the neuron’s dendritic tree but they only provide sparse and limited spatio-temporal analysis (Markram et al. 1997; Stuart et al. 1997; Perin and Markram 2013).

Aside from neurons, there are also glial cells that regulate the activity within neuronal circuits. Glial cells regulate the supply of nutrients as well as the recycling of chemicals necessary for neurons to facilitate neurotransmission. Neuronal activity is relayed by glial cells to blood vessels in order to coordinate oxygen and glucose delivery (Howarth 2014). Glial cells therefore link neurons with the cerebral vascular network and the relationship between blood flow (vascular dynamics) and neuronal activity (neurovascular coupling) can be analyzed using blood oxygen level-dependent (BOLD) functional magnetic resonance imaging (fMRI) (Ogawa and Lee 1990; Ogawa et al. 1990a; Ogawa et al. 1990b). While the temporal resolution (6–8 s) provided by fMRI cannot probe single-neuron activity, it can nonetheless analyze the dynamics of assemblies of neurons in certain brain regions. The fMRI is therefore among the pioneering techniques that has provided significant insights on the spatio-temporal dynamics of neuronal assemblies in the brain (Logothetis 2003; Jonckers et al. 2015).

**Fig. 1:**
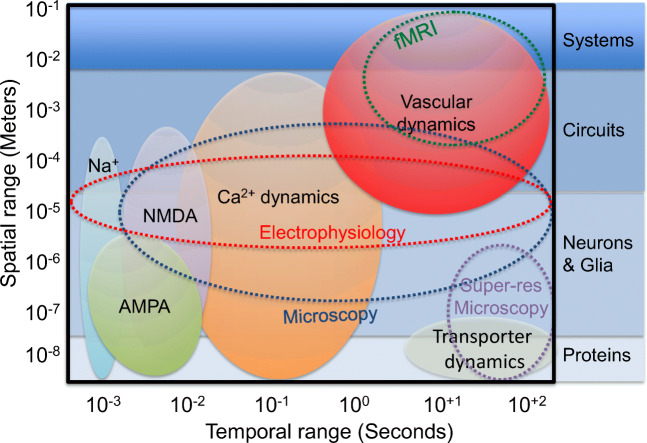
Spatio-temporal parameters and neurotechnologies for understanding the brain

Between the fast and slow acquisition times of electrophysiology and fMRI, respectively, new microscopy techniques have emerged to probe the spatio-temporal dynamics of neuronal assemblies. Figure 1 shows an overview of the various components that contribute to the spatio-temporal dynamics of brain activity with the *y*-axis showing the spatial range from nanometer to centimeter, while the *x*-axis shows the temporal range from milliseconds to minutes. The range of scales corresponds to brain regions or systems, neuronal circuits, individual cells, such as neurons and glia, and down to membrane proteins or ion channels. While ion channels have spatial dimensions in the order of nanometers, their spatio-temporal activity extends throughout the entire neuron and beyond (e.g., neuronal circuits). Taken all together, the broad spatial range makes it impossible to probe the brain in its entirety. Hence, several instruments are necessary to understand different aspects of brain function.

While electrophysiology provides good temporal resolution, optical methods using a microscope to record neuronal activity have increasingly been preferred due to their potential to provide the necessary spatial information. Optical recording via functional calcium imaging works by measuring the intracellular Ca^2+^ concentration using a fluorogenic Ca^2+^ indicator. The detected fluorescence intensity changes with Ca^2+^ concentration, which is highly correlated with neuronal activity. Functional calcium imaging offers high signal sensitivity but does not accurately represent the temporal dynamics of the membrane potential. An alternative to calcium imaging is voltage imaging, which records the changes in the membrane potential. Voltage imaging, however, has low sensitivity due to the thin membrane (~ 4 nm), which limits the number of voltage-sensing molecules to be packed in the region. Nonetheless, functional voltage imaging can be used to record fast neuronal spikes, which is not possible with calcium imaging.

Recording the changes in fluorescence following changes in the intracellular Ca^2+^ concentration or modulations in the membrane potential requires microscopes with sufficient spatio-temporal resolution. To measure these fluctuations in fluorescence, time-course images need to be acquired. The sampling time required to acquire the images should be faster than the neuronal response to satisfy the Nyquist sampling criterion. On top of that, microscopic brain structures associated with the fast temporal responses need to be spatially resolved. The current challenge is to develop a volumetric optical imaging technique that can image through living mammalian brains with high spatio-temporal resolution. 

With genetically expressed calcium and voltage indicators, we can now image the neuronal network activity in a living animal. However, optical techniques still lag and matching technologies have yet to find ideal solutions that allow us to efficiently decode the inner workings of the brain. Up to now, knowledge gaps exist between behavior, macroscopic neuronal circuit events and microscopic dendritic activity. While some works have linked macroscopic observations from *in vivo* experiments with *in vitro* dendritic activity (Petersen 2019), the sparse observations have yet to form clear and established theories on how our brain works. We are just scratching the surface, both literally and figuratively. As we study brain circuits more deeply and investigate more complex brain functions (e.g., intelligence, cognition, learning, and memory), more intricate circuit functions become apparent. Hence, decoding the inner workings of the living mammalian brain remains an incessant challenge.

The challenges for the immediate future are: (1) to combine optical stimulation and voltage imaging at multiple locations and over a physiological time scale; (2) to apply techniques now used in *in vitro* brain slices to *in vivo* studies; and (3) to increase the penetration depth of the optical recording to investigate neurons located in deep layers of the rodent brain (e.g., hippocampus). Meeting these challenges could allow us to investigate the dynamics and performance of small networks of neurons, resulting in a better understanding of the information processing in an intact living rodent brain.

In this review, we summarize the development of various optical techniques for probing neuronal circuits. To match an optical imaging approach to an experiment, we first provide a brief overview of the biophysics of neuronal responses and establish their relevance to brain function. We focus on responses from single neurons and assess the spatio-temporal characteristics of these responses. Next, we describe various optical techniques to probe these responses and discuss how these techniques differ in configuration and function. We start with the most common approach of using a wide-field epifluorescence microscope to more sophisticated imaging modalities such as holographic multi-site multi-photon microscopes. We then highlight some applications of these techniques to neuroscience and point to future challenges in decoding the mammalian brain. Beyond the topics discussed in this review are optical stimulation techniques (Go and Daria 2017; Kandori 2020), which comprise the other exciting side of neurotechnologies and optogenetics that enable probing of brain activity. Taken all together, these techniques embody the ongoing revolution in neurophotonics, which have provided major breakthroughs that pave the way to fully understand the brain.

## Biophysics and temporal response of neurons

**Fig. 2:**
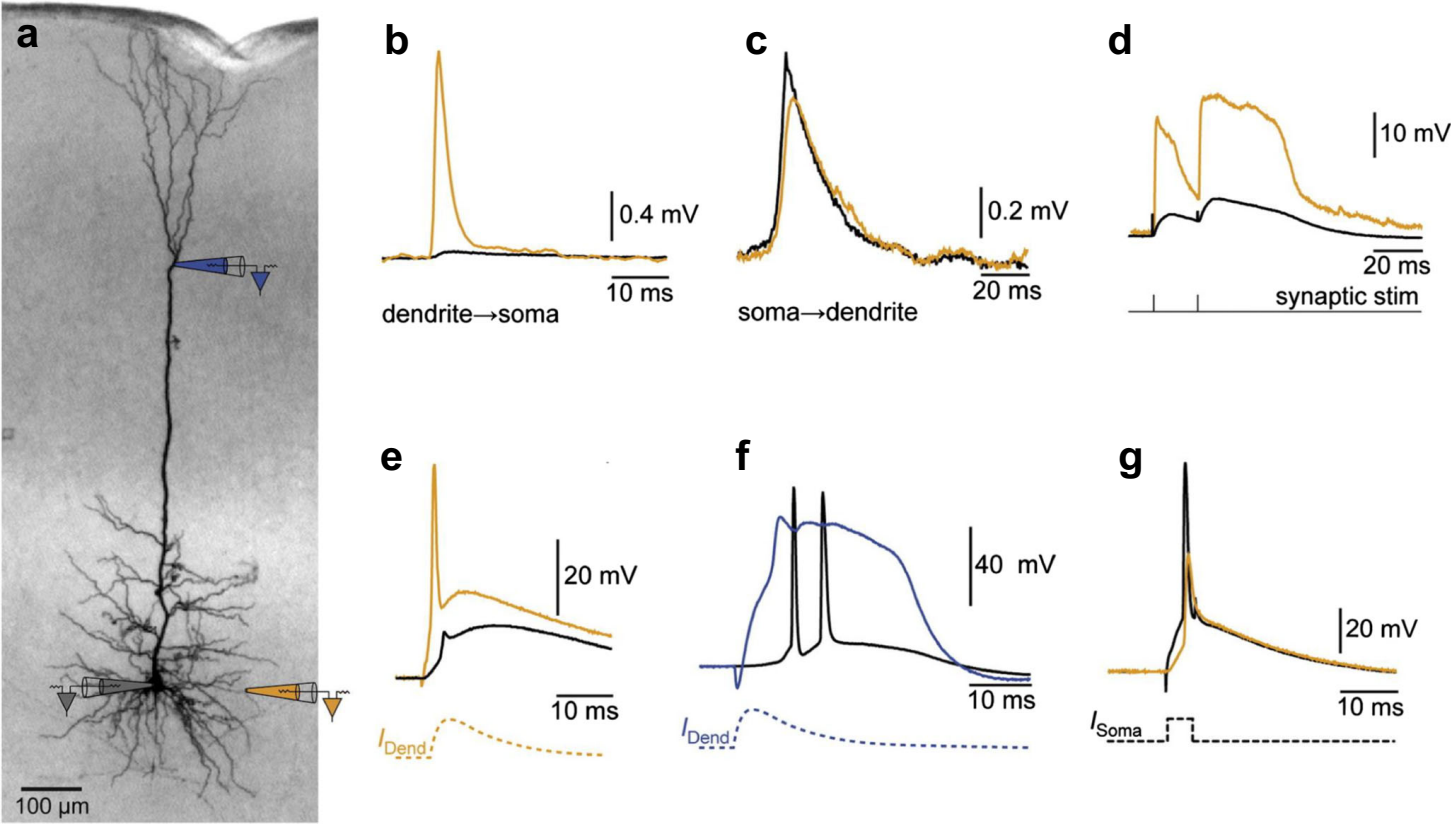
Adapted and modified with permission from Larkum and Nevian (2008). **a** Image of a biocytin-stained layer 5 pyramidal neuron annotated with positions of three electrodes at the soma (gray); the nexus of the apical tuft dendrites (blue); and a basal dendrite (orange). **b**–**g** Neuronal responses where black traces show somatic recordings while orange and blue traces correspond to dendritic recordings from electrodes identified in **a**. **b** An EPSP initiated at the basal dendrite and corresponding recording at the soma. **c** An EPSP initiated close to the soma and recorded at the basal dendrite. **d** An NMDA spike with extracellular synaptic stimulation. An EPSP-like current injection at the basal dendrite and apical nexus can generate a sodium spike (**e**) and a Ca^2+^ spike (**f**), respectively. **g** A current pulse injection at the soma produces an action potential, which can backpropagate to the dendrites

Let us outline the basic components of the neuron and identify the major temporal responses that shape its function. The neuron is bounded by a plasma membrane, which separates ions (e.g., Na^+^, K^+^, Ca^2+^, and Cl^−^) from outside and inside the neuron. When the neuron receives a synaptic input, neurotransmitters like glutamate bind to α-amino-3-hydroxy-5-methyl-4-isoxazolepropionic acid (AMPA) receptors, which are transmembrane proteins or ion-channels that gate Ca^2+^, Na^+^, and K^+^ ions into the neuron. The entry of ions into the neuron causes a transient excitatory post-synaptic potential (EPSP) that lasts for about ~ 20 ms when measured at the soma. An EPSP is typically measured using a whole-cell patch clamp. Figure 2 is adapted and modified (with permission) from Larkum and Nevian (2008), showing a layer 5 pyramidal neuron (Fig. 2a) and representative neuronal responses measured via patch electrodes at the soma (black), basal dendrite (yellow), and the nexus of the apical tuft dendrites (blue). Figure 2b shows an EPSP initiated at the dendrite and measured at the soma. On the other hand, Fig. 2c shows an EPSP initiated close to the soma and back propagated back to the basal dendrite.

Aside from the AMPA receptor, there are ion-channels that open up when the membrane potential reaches a certain threshold. The N-methyl-d-aspartic acid (NMDA) receptor is a ligand-gated ion-channel triggered by neurotransmitters (e.g. NMDA, glutamate or glycine). However, unlike AMPA, the NMDA receptor is also voltage dependent due to the presence of magnesium that blocks the channel. To activate NMDA, several AMPA-driven EPSPs are required to depolarize the neuron and remove its magnesium block. Once the block is removed, a non-linear change in the membrane potential generates an NMDA spike (see Fig. 2d), which is a common dendritic spike observed in thin dendrites such as the basal and apical tuft dendrites (Schiller et al. 2000). NMDA spikes can last for about ~ 50 ms (Larkum and Nevian 2008). Dendritic spikes are also produced by other voltage-gated ion-channels such as fast Na^+^ spikes (Fig. 2e) that lasts ~ 2 ms and Ca^2+^ spikes (Fig. 2f) that lasts ~ 40 ms.

Multiple synaptic inputs and dendritic spikes build-up along different regions of the neuron to reach a threshold to fire an AP (Fig. 2g). An AP is a fast spike (~ 1 ms) with temporal response shaped by Na^+^ and K^+^ channels. During an AP, Ca^2+^ ions also enter via voltage-gated calcium channels (VGCCs) and NMDA receptors among others (Catterall 2000; Clapham 2007; Grienberger and Konnerth 2012). An AP also triggers the transmission of neurotransmitters to neighboring neurons via synapses. An AP can also backpropagate towards the neuron’s dendritic tree (Fig. 2g) and can induce the generation of dendritic spikes (Stuart et al. 1997; Larkum and Nevian 2008).

## Photosensitive molecules and proteins for recording neuronal activity

### Molecular indicators

Calcium imaging makes use of fluorogenic Ca^2+^ indicators that reports the concentration of intracellular Ca^2+^ can bind to (Grienberger and Konnerth 2012). Neuronal activity changes the intracellular Ca^2+^ concentration, which in turn changes the intensity of the emitted fluorescence. Imaging the changes in fluorescence due to changes in intracellular Ca^2+^ concentration offers a high sensitivity however, there are other sources that modulate intracellular Ca^2+^ concentration and therefore do not accurately correspond to the cell’s electrical activity. Moreover, intracellular Ca^2+^ dynamics are slow and therefore not fast enough to characterize fast electrical activity such as APs. Nonetheless, sufficient information on circuit activity can still be deduced. Functional calcium imaging has been used to study dendritic activity in single neurons (Tank et al. 1988; Yuste and Katz 1991; Yuste et al. 1994), providing evidence for the existence of Ca^2+^ electrogenesis in the distal apical dendrites of cortical pyramidal neurons (Schiller et al. 1997; Larkum et al. 1999).

Tsien (1980) synthesized the first Ca^2+^ indicator, which was modified from the selective Ca^2+^ chelator ethylene glycol-bis(β-aminoethyl ether)-*N,N,N’N’*-tetraacetic acid (EGTA) to form 1,2-bis(*o*-aminophenoxy) ethane-*N*,*N*-*N*’,*N*’-tetraacetic acid (BAPTA). At that time, the major requirements for the design of the indicator were to make it selective between Mg^2+^ and Ca^2+^ as well as to improve its capacity to be loaded into cells without disrupting the membrane (acetoxymethyl (AM) esters) (Tsien 1981; Tsien et al. 1982). The next generation of Ca^2+^ indicators was based on Fura-2 (aminopolycarboxylic acid), which greatly improved the fluorescence yield by 30-fold by a larger blueshift in the excitation/emission spectra with increasing Ca^2+^ concentration (Grynkiewicz et al. 1985). However, the application was limited since Fura-2’s excitation wavelength is in the ultra-violet. Later on, visible-wavelength indicators based on rhodamine (Rhod-2) and fluorescein (Fluo-2/Fluo-3) were developed and are now commonly available (Minta et al. 1989). Another recently developed BAPTA-based Ca^2+^ indicator is Cal-520, which has a better signal-to-noise ratio (SNR) and faster decay time (0.2-s slow component) compared to Oregon Green can BAPTA-1 (OGB-1) (Tada et al. 2014). Cal-590, a redshifted version of Cal-520, has been successfully used to image Ca^2+^ activity of layer 5/6 pyramidal neurons *in vivo* (Tischbirek et al. 2015).

Voltage imaging works by sensing the changes in the electric field across the thin plasma membrane (~4 nm) of neurons. The membrane, separating the extracellular medium and the cytoplasm, acts as a capacitor that sets up an electric field across the membrane (Olivotto et al. 1996). The earliest form of optical sensing of the membrane potential in nerve fibers was initially observed from the changes in optical properties (light scattering, birefringence, and fluorescence) of the membrane during an action potential (Cohen et al. 1968; Tasaki et al. 1968). Salzberg et al. (1973) later used a merocyanine dye, which was the first voltage sensitive dye (VSD) used for detecting action potentials in sensory neurons of leech segmental ganglia. Changes in the ion concentration in the cell modulates the electric field across the membrane and induces a change in the spectral properties of VSDs bound to the membrane. By using an improved VSD, merocyanine-oxazolone, a larger signal was obtained enabling simultaneous optical recording from eight neurons (Grinvald et al. 1977).

The practicality of using VSDs is limited by their low sensitivity, which is primarily due to the finite surface area where VSDs are bound. The strength of the electric field decreases exponentially with distance from the membrane and VSDs need to be bound to the plasma membrane to effectively sense the changes in the electric field. Moreover, a large number of VSDs bind to other intracellular structures, which emit a relatively strong background fluorescence that do not change with the membrane potential. Apart from the low sensitivity and strong background fluorescence, excited VSDs generate reactive oxygen species, which can be toxic to the cell.

The sensitivity and response times of VSDs vary depending on the mechanisms that sense the membrane potential (Peterka et al. 2011). A mechanism based on reorientation of the VSD’s dipole moment with the electric field can provide fast sensing of changes in the membrane potential (Dragsten and Webb 1978). The reorientation of the dipole moment changes the interaction of the VSD with the excitation light and consequently alters the fluorescence spectra. A mechanism referred to as redistribution works by rapid potential-dependent repartitioning of VSDs on the cell membrane and the nearby aqueous salt solution (Sims et al. 1974; Ehrenberg et al. 1988). The transient change of concentration of dye molecules bound to the membrane changes the efficacy of light absorption. On the other hand, hemicyanine VSDs (e.g. JPW-1114 or its variant JPW-3028) exhibit electrochroism, which can probe fast changes in membrane potential (Loew et al. 1985; Antic and Zecevic 1995; Zecevic 1996; Antic 2003; Yan et al. 2012; Loew 2011). In addition, electrochromic VSDs such as ANNINE-6plus (Fromherz et al. 2008) and fluorinated-hemicyanine dyes (Yan et al. 2012) have been used with two-photon (2P) excitation. Using the fluorinated-hemicyanine dye with single-voxel recording at 10 kHz, Acker et al. (2011) were able to measure back propagating APs invading dendritic trees with sensitivities of more than 16% per 100 mV using a 1060 nm 2P excitation wavelength.

Compared to VSDs, Ca^2+^ indicators are preferred for optical recording of neuronal activity because of its high sensitivity and SNR. However, as discussed earlier, calcium imaging do not accurately report the neuron’s electrical activity as there are many sources of Ca^2+^ such as those from internal Ca^2+^ stores. Moreover, the slow changes in Ca^2+^ concentration makes it difficult to probe bursts of action potentials. Nonetheless, both voltage and calcium imaging techniques offer complementary insights when used to analyze neuronal activity (Berger et al. 2007; Roome and Kuhn 2018).

### Genetically encoded indicators

In 1997, Roger Tsien’s group revolutionized calcium imaging by developing genetically encoded Ca^2+^ indicators (GECIs), which they refer to as “Cameleons” (Miyawaki et al. 1997). Cameleons consist of a calcium-modulated protein or Calmodulin (CaM) and a pair of spectrally overlapping mutants of green fluorescent proteins (GFPs) that are excited via Förster resonance energy transfer (FRET). Binding of Ca^2+^ increases the FRET between the GFP-pair, resulting in fluorescence emission that can be correlated with intracellular Ca^2+^ concentration. Since then, several improvements have been proposed (Whitaker 2010), including the use of a single GFP Ca^2+^ probe, which is now commonly known as GCaMP (Nakai et al. 2001). GCaMP indicators have undergone several improvements, and recent versions (i.e., GCaMP6s and GCaMP6f) are now widely used in experiments *in vivo* (Tian et al. 2009; Akerboom et al. 2012; Chen et al. 2013). These GECIs can be expressed in cells using Adeno-associated virus (AAV) injection (Tian et al., 2009) or transgenic mouse lines (Heim et al. 2007; Zeng and Madisen 2012). GECIs are useful in recording calcium activity at the cell bodies and dendrites while the animal is receiving sensory inputs or performing a behavioral task (Xu et al. 2012; Palmer et al. 2014).

On the other hand, genetically encoded voltage indicators (GEVIs) have been demonstrated to work on cell cultures, slices, and *in vivo* (Kralj et al. 2012; St-Pierre et al. 2014; Gong et al. 2014; Hochbaum et al. 2014; Gong et al. 2015; Carandini et al. 2015). Most GEVIs also operate via FRET, where they undergo a conformational change with modulations in the membrane potential. GEVIs are synthesized by inserting a pair of FRET fluorescent proteins into voltage-sensing transmembrane segments of voltage-gated potassium channels (Sakai et al. 2001). GEVI variants have yielded higher sensitivity and fast temporal response (Akemann et al. 2012; St-Pierre et al. 2014). The archaerhodopsin-3 (Arch) is a GEVI that offer high sensitivity and fast recording. Arch has an absorption peak at 558 nm and emits 687-nm fluorescence. The sensitivity of Arch is roughly 4% with a sub-millisecond (< 0.5 ms) response time (Kralj et al. 2012; Hochbaum et al. 2014). While GEVIs have promising applications for recording membrane potentials, several issues remain to be optimized such as: (1) speed (slow temporal response (~ 1 ms) compared to VSDs with 0.001 ms temporal resolution); (2) linearity (exhibit a non-linear sigmoid response with membrane voltage between − 100 and 100 mV); (3) have limited spectral range of excitation and emission; and (4) increased membrane capacitance (Antic et al. 2016).

Light-gated ion-channels (or Rhodopsins) comprise the other side of neurotechnologies that have not been discussed in this review. A recent review by Kandori (2020) discusses the biophysics of photoreceptive proteins and their relationship to optogenetics. Combining rhodopsins and GEVIs in a single construct allows for simultaneous stimulation and read-out of membrane activity. The light-activated Quasar 3 (paQuasAr3), a mixture of QuasAr2 opsin-based GEVI and Channelrhodopsin CheRiff, can be excited by blue light to activate CheRiff and record the activity from the QuasAr2 (Adam et al. 2019). 

## Optical imaging of neuronal activity

**Fig. 3:**
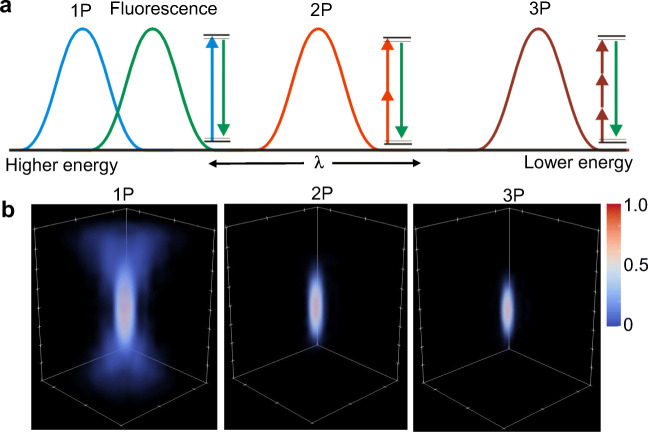
**a** Energy diagram and excitation spectra for single-photon (1P), two-photon (2P), and three-photon (3P) absorption and corresponding fluorescence emission spectrum (green). **b** 3D point spread function of the normalized fluorescence intensity via 1P, 2P, and 3P excitation

To optically record neuronal activity from the changes in fluorescence from either molecular or genetically encoded indicators, two modes of excitation are available: linear single-photon (1P) excitation and non-linear multi-photon excitation, which can either be two-photon (2P) or three-photon (3P) excitation. Figure 3a shows a representative spectral diagram relating the emitted fluorescence (green) via 1P (blue), 2P (red), and 3P (brown) excitation. A linear 1P excitation requires photons with high energy (typically in the visible region) to excite molecules/proteins to emit fluorescence. In this case, the fluorescence intensity is linearly proportional to the intensity of the excitation light. Due to the linear process of 1P excitation, the absorption process is highly probable and fluorescence is emitted wherever a dye molecule absorbs an incident photon. Figure 3b (left) shows the 3D point spread function of the normalized fluorescence intensity via 1P excitation. Illumination for 1P excitation is less demanding and can use commonly available light sources such as mercury lamps, light-emitting diodes, and low-power continuous-wave lasers.

On the other hand, non-linear multi-photon excitation requires *n* low-energy photons (typically in the near infrared) to be simultaneously absorbed by a dye molecule (Goeppert-Mayer 1931). The emitted fluorescence intensity is related to the excitation intensity given by, 〈*F*〉~*σ*_*n*_*I*^*n*^, where *σ*_*n*_ is the multi-photon cross section of the dye molecule and *n* is the order of excitation which can be *n* = 2 for 2P or *n* = 3 for 3P. Figure 3b shows the 3D point spread function of the normalized fluorescence intensity via 2P (middle) and 3P (right) excitation. The probability of multi-photon absorption is very low, thereby requiring special conditions on the focusing optics as well as the excitation laser. To increase the probability, the density of excitation photons in space and in time must be sufficiently high. Using an objective lens with high-numerical aperture brings about a tight focus and consequently confines the probability of fluorescence emission within the diffraction-limited focal volume (~ 0.1 μm^3^) (Zipfel et al. 2003). Moreover, increasing the photon density within an ultrashort laser pulse (~ 100 fs) increases the probability of multi-photon excitation to occur within the short laser pulse while maintaining a low average power.

The differences between 1P versus multi-photon excitation entail appropriate light sources and optical designs to acquire the images. Optical designs consider the differences in spectral properties (Fig. 3a) to allow proper filters and dichroic mirrors to separate the fluorescence from the excitation light. In the succeeding section, we describe various optical setups that can be used to image neuronal activity using the photochemical and optogenetic tools described in the previous section.

### Single-photon wide-field and confocal microscope

**Fig. 4:**
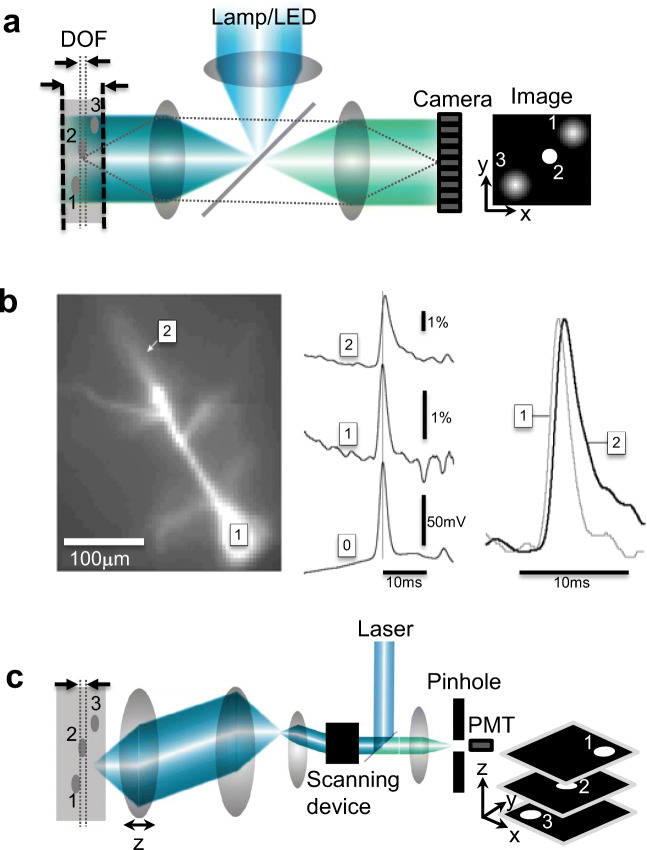
**a** A 1P epifluorescence microscope. Relative axial discrimination is shown in dashed lines for **a** and **c**. Inset shows the representative 2D image output. **b** Adapted with permission from Antic (2003). (Left) A 1P image of a layer 5 pyramidal neuron loaded with JPW-3028 showing the soma and proximal apical dendrites. (Middle) Somatic whole-cell recording (0) and optical recording at the (1) soma and (2) apical trunk. (Right) Scaled optical recordings at the soma (gray) and apical dendrite (black) for direct comparison of the timing and shape of the signals. **c** A 1P laser scanning confocal microscope with de-scanned detection. Inset shows the representative 3D image output

Functional imaging of neuronal activity can be achieved using a conventional epifluorescence microscope (Fig. 4a) and a two-dimensional (2D) multi-channel image sensor (or camera) for video acquisition (Connor 1986; Lasser-Ross et al. 1991). However, epifluorescence microscopes use 1P excitation and dye molecules readily emit fluorescence where an excitation photon exists. Fluorescence emitted from off-focus planes appear blurred when acquired by the camera thereby limiting the use of such microscopes to thin brain slices (~ 10 μm) or cell cultures. When imaging the activity of neurons within thick (~ 300 μm) brain slices or from an intact brain (*in vivo*), structures outside the depth-of-focus (DOF) also appear blurred, which degrades the contrast and overall image quality. A representative image in Fig. 4a shows blurred images of off-plane structures #1 and #3, while in-plane structure #2 appears focused.

Wide-field microscopes using highly sensitive cameras can acquire images as fast as ~ 1000 frames/s. Cameras built with complementary metal oxide semiconductor (CMOS) light sensors or electron-multiplying charged coupled device (EMCCD) offer high-speed and highly sensitive imaging. EMCCD cameras have high sensitivity (i.e., ~ 90% quantum efficiencies), large dynamic range, and high sampling rates (up to 4000 frames/s). Using wide-field epifluorescence microscope to image neurons loaded with VSDs allows imaging of the membrane potential (Antic 2003; Foust et al. 2010). Figure 4b is adapted (with permission) from Antic (2003), which shows the activity at the soma, basal, and apical oblique dendrites of a layer 5 pyramidal neuron loaded with VSD (JPW-3028). This technique has been successful in an optical recording of subthreshold and suprathreshold membrane potential at the axon initial segment (Palmer and Stuart 2006; Foust et al. 2010; Popovic et al. 2011), soma (Berger et al. 2007), apical trunk, thin oblique and basal dendrites (Antic 2003; Zhou et al. 2008; Holthoff et al. 2010; Zhou et al. 2015), and dendritic spines (Palmer and Stuart 2009; Popovic et al. 2014). The same technique has also been used to record action potentials using a GEVI expressed in cultured mammalian neurons (Kralj et al. 2012) and probe Ca_2+_ activity in an *in vitro* neuronal network expressing GECI (GCaMP6m) (Marom et al. 2016).

Combining VSDs and Ca^2+^ indicators allows simultaneous optical recording of the membrane potential and Ca^2+^ activity as long as their emission spectra do not non-overlap but with slightly overlapping absorption spectra (Berger et al. 2007)). For example, 1P excitation of a VSD (JPW-1114) and calcium indicator (calcium green) can be achieved using light with 488-nm wavelength while an appropriate dichroic mirror can be used to distinguish the emitted fluorescence from the two indicators (Bullen and Saggau 1998).

It is important to discriminate fluorescence photons emitted from different axial planes to improve the contrast especially when structures overlap along the *z*-axis. To isolate fluorescence photons within a single plane, a confocal microscope can be used. In a confocal microscope, fluorescence outside the focal plane is discriminated via a pinhole before it gets detected using a single-channel detector such as a photomultiplier tube (PMT) or an avalanche photodiode. Figure 4c illustrates a basic confocal configuration, where a 2D beam scanner is built with a pair of galvanometer mirrors (GM) to scan the focus and render a 2D image along the focal plane. The emitted fluorescence is detected back through the scanning mirrors and onto the pinhole and a detector. The numerical aperture of the lens and the size of the pinhole define the depth-of-focus (DOF) or thickness of the imaging plane. A representative image in Fig. 4c shows high-contrast images of the sample can be taken from multiple optical sections by translating (along *z* or optical axis) either the sample or the objective lens. Using GM scanner, an image with 1000 × 1000 pixels can be rendered in ~ 1 s (or 1 frame/s), while a 500 × 500-pixel image can be rendered in ~ 0.25 s (or 4 frames/s).

A faster scanning method is achieved using a resonant scanning mirror along the *x*-axis and a galvanometer mirror along the *y*-axis. A resonant mirror with a fixed rate of ~ 10 kHz equates to 20,000 lines per second (i.e., 2 lines per period of oscillation). Hence, an image with 1000 lines can be acquired in ~ 50 ms (or 20 frames/s). The number of acquired pixels along the *x*-axis will depend on the speed of the photodetector and the data acquisition system. To create a 1000 × 1000-pixel image, the sensor should be able to acquire the fluorescence within ~ 50 ns. Faster frame rates can be achieved by reducing the number of lines (e.g., a 500-line image can be acquired in ~ 25 ms). Hence, the speed of scanning and the size of the region-of-interest scanned define the temporal resolution of the system. Confocal microscopes have been used for functional calcium imaging in a range of preparations (Smith and Augustine 1988; Williams and Fay 1990; Eilers et al. 1995; Knight et al. 2003).

**Fig. 5:**
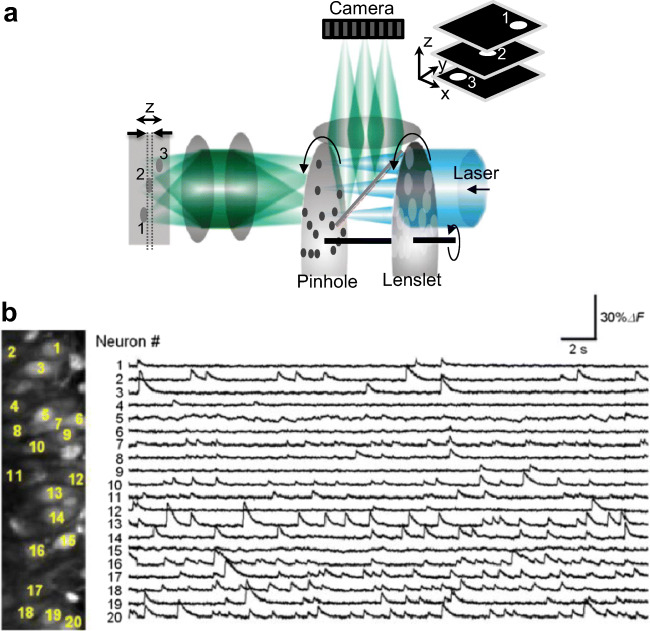
**a** Nipkow disk confocal microscope. Relative axial discrimination is shown in dashed lines. Inset shows the representative 3D image output. **b** Adapted with permission from Takahashi et al. (2010). (Left) Twenty neurons were monitored at 2000 frames/s. (Right) Spontaneous Δ*F*/*F* traces of individual neurons; the locations were shown in the left image

High-speed confocal microscopes make use of scanning Nipkow disks (Takahashi et al. 2011; Takahara et al. 2011; Sakurai et al. 2014) and parallel illumination using diffractive optical elements (Krmpot et al. 2019) (Fig. 5a). Krmpot et al. (2019) used a diffractive optical element to simultaneously excite the sample in a 32 x 32 foci array and detected the fluorescence from each focus in a confocal arrangement using a matching detector array comprising of avalanche photodiodes. A high-speed 1P confocal microscope using a Nipkow disk and an EMCCD can also be used for high-speed calcium imaging of a large population of neurons. Takahashi et al. (2010) used the technique to study spike synchronization in hippocampal neuronal networks. Figure 5b is adapted (with permission) from Takahashi et al. (2010), which shows Ca^2+^ responses from 20 neurons loaded with OGB-1 AM and acquired at 2000 frames/s via a Nipkow disk confocal microscope.

### Light-sheet microscope

**Fig. 6:**
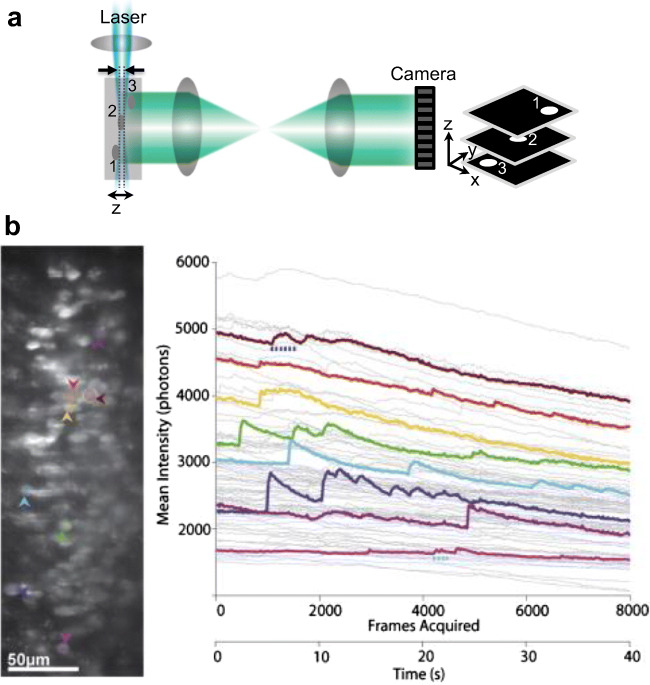
**a** A 1P light-sheet microscope. Relative axial discrimination is shown in dashed lines. Inset shows the representative 3D image output. **b** Adapted with permission from Holekamp et al. (2008). (Left) Image of vomeronasal sensory neurons. (Right) Time courses of the fluorescence intensity neurons in the area imaged. Intensity traces from the subset of cells marked in (A) are coded by color

With EMCCD and sCMOS cameras, high-speed and highly sensitive image acquisition can be performed. To improve the contrast and optical sectioning, a widefield microscope can be constructed with illumination by a sheet of light along the imaging plane (Fig. 6a). This technique is referred to as the “light-sheet” microscope, which was originally called orthogonal-plane fluorescence optical sectioning microscopy (Voie et al. 1993) and later as selective plane illumination microscopy (SPIM) (Huisken et al. 2004; Huisken and Stainier 2009). SPIM can use a light sheet (generated via a cylindrical lens) or a one-dimensionally scanned loosely focused beam of excitation light that is directed orthogonal to the imaging axis. A non-diffracting Bessel beam can also be scanned across the sample to generate a light sheet (Fahrbach and Rohrbach 2010; Fahrbach et al. 2013b; Corsetti et al. 2019). The excitation is typically via 1P excitation and illuminates the planar section of the sample. However, 2P (Fahrbach et al. 2013a) and 3P (Escobet-Montalban et al. 2018) light sheet with a Bessel beam has also been demonstrated.

Since the excitation is localized within a plane, light-sheet microscopes can perform optical sectioning while imaging at high speed using a camera. Depending on the resolution of the camera’s region-of-interest (ROI), high-speed volumetric imaging is possible and the activity of a large population of neurons can be recorded in semi-transparent animals such as the zebrafish (Ahrens et al. 2013; Panier et al. 2013; Hammen et al. 2014; Wolf et al. 2015). Recent improvements focus on increasing the temporal resolution, spatial resolution, and the possibility for its utilization in non-transparent tissues (Taylor et al. 2018).

Variants of the light-sheet technique allow for *in vivo* imaging of neuronal activity in the intact mouse brain (Holekamp et al. 2008; Engelbrecht et al. 2010; Bouchard et al. 2015). Holekamp et al. (2008) re-engineered the microscope to couple the light-sheet illumination with the detection objective lens in order to study pheromone-sensing neurons of the mouse vomeronasal organ *in vivo*. Figure 6b is adapted (with permission) from Holekamp et al. (2008) showing vomeronasal sensory neurons of the intact mouse brain. The neurons were labeled with the calcium indicator OGB-1 by pressure-injection of the dye via a tube inserted through the nasal pathway. The field-of-view was imaged at 200 frames/s and recorded the changes in the fluorescence due to spontaneous activity of the cells.

**Fig. 7:**
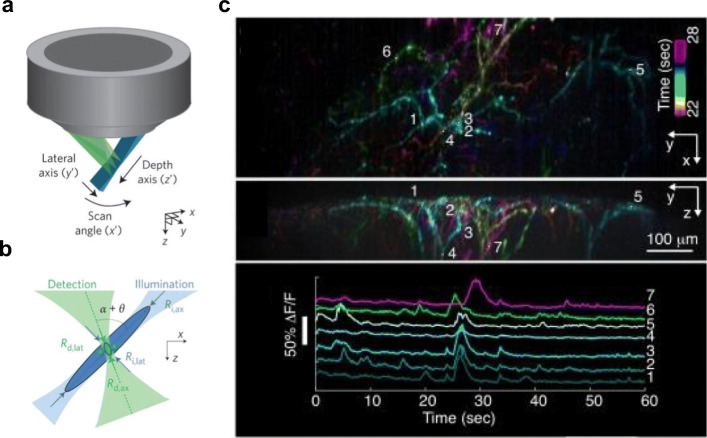
**a**, **b** Adapted with permission from Bouchard et al. (2015). SCAPE microscopy: planar excitation using a single objective lens. **b** Orthogonally aligned illumination and detection point spread function for SCAPE microscopy. **c** Adapted with permission from Hillman et al. (2018). Images of 5 neurons in the whisker barrel cortex. (Bottom) Calcium response from the 5 neurons

On the other hand, the variant developed by Bouchard et al. (2015) is referred to as swept confocally aligned planar excitation (SCAPE) microscope. Figure 7a is adapted (with permission) from Bouchard et al. (2015) where a GM scanner was used to sweep an obliquely directed light sheet laterally across the sample, while maintaining the focus of the camera on the moving plane. The technique allows for light-sheet volumetric imaging through a single, stationary objective lens, and capable of volumetric imaging at 10 volumes per second. The detection camera is obliquely aligned to focus onto the obliquely directed light sheet through the sample. Figure 7b is adapted (with permission) from Hillman et al. (2018), which shows calcium imaging traces (bottom) of spontaneous activity in the apical dendrites of layer 5 neurons expressing GCaMP6f (top) in the whisker barrel cortex of an awake, behaving mouse acquired via the SCAPE microscope.

### Time-multiplexed scanning two- and three-photon imaging

**Fig. 8:**
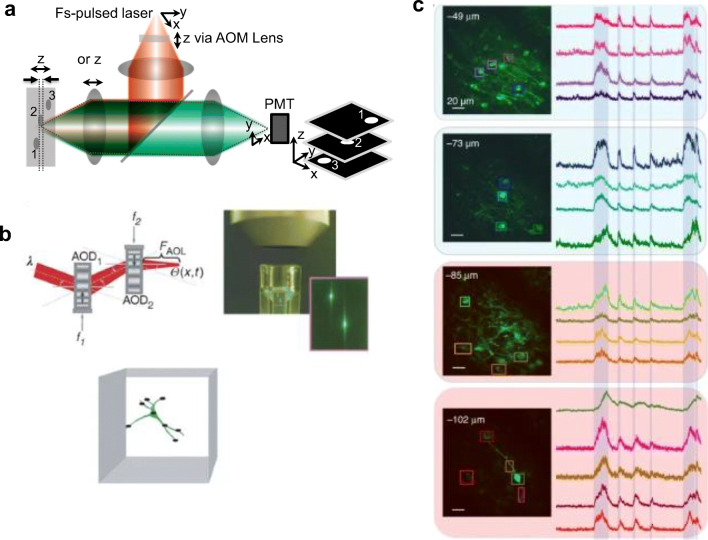
**a** A 2P microscope using different modes of axial scanning by the sample, objective lens, or via an acousto-optic modulator (AOM). 2P microscope can either use beam scanners to scan along the focal plane via galvanometer mirrors, resonant mirrors, and AOMs. Relative axial discrimination is shown in dashed lines. Inset shows the representative 3D image output. **b** Adapted with permission from Reddy et al. (2008). AOM-based 3D scanner. **c** Adapted with permission from Nadella et al. (2016). (Left) Images of layer 2/3 pyramidal neurons of the visual cortex expressing GCAMP6f. (Right) Corresponding calcium responses

The laser scanning 2P microscope (Fig. 8a) has revolutionized calcium imaging in addition to providing 3D images of neuronal circuits (Denk et al. 1990; Svoboda et al. 1997; Helmchen and Denk 2005). 2P microscopes have also been used for voltage imaging (Kuhn et al. 2008; Fisher et al. 2008; Acker et al. 2011; Yan et al. 2012; Ahrens et al. 2012; Akemann et al. 2013b; Akemann et al. 2013a; Roome and Kuhn 2018; Wu et al. 2020). The fluorescence from 2P excitation is collected by a single-channel detector such as a PMT or an avalanche photodiode. With a conventional GM scanner, the focus of the excitation laser is raster scanned to render an image along the focal plane. However, as with 1P scanning confocal microscopes, the scanning speed can be increased using a resonant mirror and GM scanner combination. With images acquired in less than 100 ms via resonant mirrors, calcium imaging experiments can be performed.

However, obtaining an image by raster scanning acquires pixels even at regions where relevant signals are unlikely to occur. If locations of the signals can be known, acquiring signals at targeted regions of the sample can be achieved without raster scanning. Such a technique is referred to as random access (RA) microscopy, which cannot be achieved using a resonant mirror due to its inability to randomly position the focus within the field-of-view. Using a pair of GM mirrors allows arbitrary positioning of a focus in 2D. Such technique has been used for time-shared optical tweezers for manipulating an array of particles (Sasaki et al. 1991). The downside for using GM mirrors when scanned arbitrarily is the slow settling time (~ 300 μs), which limits the temporal resolution. In contrast, raster scanning using GM scanners involves small angle movements and settling time can be within ~1 μs for small angle mirror movements.

An acousto-optic modulator (AOM) has a faster settling time (~ 10 μs) even at large-angle movements and a non-mechanical alternative compared to GM scanners. They can be used to scan randomly to allow for patterned illumination and have also been initially used for optical manipulation of multiple particles (Visscher et al. 1996) and later for multi-site calcium imaging (Reddy and Saggau 2005; Iyer et al. 2006). Using two AOMs facilitates high-speed random focal spot positioning within 2D. A 3D beam scanning system using four AOMs was proposed (Reddy et al. 2008; Kirkby et al. 2010) and now currently used for calcium imaging (Katona et al. 2012). Figure 8b is adapted (with permission) from Reddy et al. (2008), which shows how AOMs deflect an incident beam via a controllable optical grating, which is formed by standing waves in a crystal induced from an applied radio-frequency signal. Scanning speeds have significantly improved with the use of AOMs and RA multi-photon microscopes are now used for optical recording of Ca^2+^ activity in neuronal networks (Reddy and Saggau 2005; Reddy et al. 2008; Katona et al. 2012; Nadella et al. 2016).

While AOMs offer the fastest response, they have low optical throughput (~ 50% per AOM). A 2D scanning system uses two cascaded AOMs with a total throughput of ~ 25%, while a 3D system uses four cascaded AOMs with a total optical throughput of ~ 6%. Moreover, when used for randomly positioning recording sites, the sampling time is shared, which sets the exposure time per site to be inversely proportional to the total number of recording sites.

Nadella et al. (2016) improved the performance by using AOMs with optimized efficiency and high-speed instrumentation to image the visual cortex in awake head-fixed mice on a cylindrical treadmill. Figure 8c is adapted (with permission) from Nadella et al. (2016) showing layer 2/3 pyramidal neurons of the visual cortex expressing GCAMP6f (left) and corresponding calcium responses (right). RA microscopy does not acquire volumetric images but acquires arbitrary voxels (3D pixel) in 3D and the system developed by Nadella et al. (2016) is capable of acquiring one voxel within 100 ns. AOMs have also been used to image GEVIs in an *in vitro* preparation (Chamberland et al. 2017) and i*n vivo* (Villette et al. 2019).

**Fig. 9:**
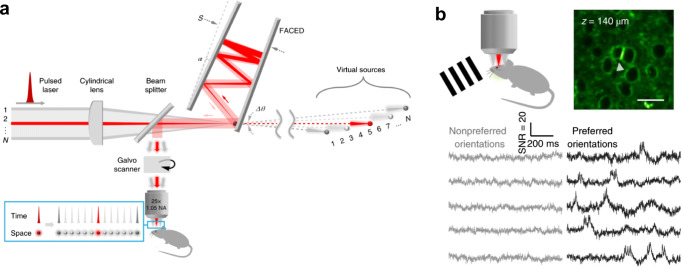
**a**, **b** Adapted with permission from Wu et al. (2020). **a** FACED technique to split a femtosecond pulsed laser into multiple sub-pulses to form a spatially separated and temporally delayed multiple foci at the sample plane. **b** Voltage traces from visual cortex (V1) neurons showing orientation selectivity. Preferred orientations (black traces) show more sub- and suprathreshold activity than nonpreferred orientations (gray traces)

Apart from the use of AOMs, several techniques have been proposed to improve the speed of raster scanning in 2P microscopy for imaging fast voltage responses. Zhang et al. (2019) scanned multiple foci to achieve a temporal resolution of up to 1000 frames/s to image calcium activity *in vivo*. Fluorescence from the multiple foci was collected using a high-speed sCMOS camera. They reduced the repetition rate and increased the pulse energy of the laser to improve the fluorescence yield per focus similar to the technique proposed by Castanares et al. (2016) for multi-site holographic imaging. On the other hand, Kazemipour et al. (2019) scanned and sampled the fluorescence from line foci projected at several angles along the sample plane to achieve high-speed imaging of up to 1016 frames/s. A technique they refer to as scanned line angular projection (SLAP) microscopy. Just recently, Wu et al. (2020) proposed a laser scanner based on a free-space angular chirp-enhanced delay (FACED) technique that can be incorporated into a standard 2P microscope. Figures 9a and 9b are adapted (with permission) from Wu et al. (2020). Figure 9a shows the FACED technique where they used a cylindrical lens and a parallel mirror to split the femtosecond pulsed laser into multiple sub-pulses to form a spatially separated and temporally delayed multiple foci at the sample plane. With the FACED technique, they can acquire images up to 1000 frames/s. Figure 9b shows representative images of neurons in the visual cortex and their corresponding voltage responses from the visual cortex (V1) neurons showing orientation selectivity.

Using an axially extended focus via the use of a Bessel beam has gained attention. While Bessel beams have been used in a laterally projected beam for light-sheet imaging (as discussed earlier), they were initially introduced in microscopy to be projected along the optical axis of an imaging system (Arimoto et al. 1992). A Bessel beam is non-diffractive and produced using an axicon lens. Such beam has been used for simultaneous optical manipulation of particles along multiple planes (Garces-Chavez et al. 2002) and has been implemented in 2P (DuFour et al. 2006; Theriault et al. 2013) and 3P (Rodriguez et al. 2018; Chen et al. 2018) microscopes. With video-rate volumetric functional imaging and high spatial resolution capable of imaging dendritic spines, it has been used for optical recording of neuronal activity.

**Fig. 10:**
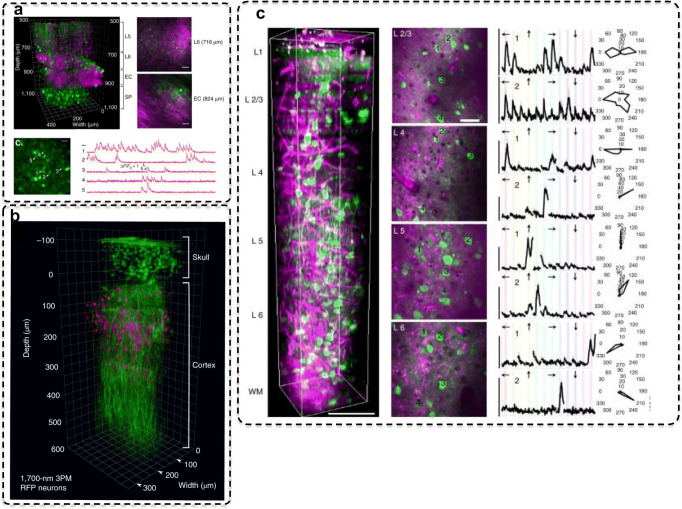
**a** Adapted with permission from Ouzounov et al. (2017). (Top left) 3D reconstruction of 3P images of GCaMP6s-labeled neurons in the mouse cortex and the hippocampus (green, fluorescence, magenta, third-harmonic generated signal). (Right top and middle) Selected XY frames at various depths in **a**. (Bottom left) Image of the *stratum pyramidale* layer of the hippocampus and (bottom right) corresponding spontaneous activity recorded from the labeled neurons. **b** Adapted with permission from Wang et al. (2018), where they used a 3P microscope to achieve functional calcium imaging through an intact skull of a rodent. **c** Adapted from Yildirim et al. (2019). (Left) Three-dimensional rendering of a sequence of 450 lateral 3P images. (Middle) Selection of lateral images from layers 2/3, 4, 5, and 6. (Right) Average calcium responses (ΔF/F) for representative cells in each layer over 10 trials

The use of 3P microscopes has recently emerged with new laser designs that emit in the infrared region (Horton et al. 2013). Compared to 2P excitation, 3P excitation provides improved optical sectioning since the probability of absorbing three photons simultaneously is much lower. Hence, fluorescence emission is more localized along the optical axis and thus improving the contrast when imaging at lower depths through the tissue. Moreover, the use of longer wavelength suffers less scattering through biological tissues enabling 3P microscopes to image deeper into the brain (~ 0.5-mm depth) (Ouzounov et al. 2017; Wang et al. 2018; Yildirim et al. 2019; Wang et al. 2020). Ouzounov et al. (2017) used a 3P microscope to image both the structure and activity of neurons expressing GCaMP6s in the hippocampus of the intact mouse brain. Figure 10a is adapted (with permission) from Ouzounov et al. (2017), which shows highly resolved neurons in depths of 500 to 1100 µm from the dura of the brain, which ranges from layers 5 and 6 of the cortex region to the external capsule and *stratum pyramidale* layers of the hippocampus. The green channel is fluorescence excited via 3P, while the magenta channel is the third-harmonic generation (THG) from other features in the tissue (e.g., blood vessels, white matter, and myelinated axons) that did not express a fluorogenic indicator. Calcium transients are also shown on identified neurons. Moreover, the same group has also succeeded in imaging through the intact skull and performed functional 3P calcium imaging in cortical layer 2/3 of an awake mice (Wang et al. 2018). Figure 10b is adapted from Wang et al. (2018), which shows a 3D reconstruction of a cortical column through the mice skull. The red channels are neurons expressing red fluorescent protein (RFP) while the green channel is THG from other features in the tissue.

A 3P microscope has also been used to study sensory responses in the visual cortex. Yildirim et al. (2019) performed functional imaging of cortical neurons expressing GCaMP6s in all layers of the visual cortex of the mice. Figure 10c is adapted from Yildirim et al. (2019), which shows the entire depth of the visual cortex showing neurons and blood vessels. As with previous works with 3P microscopes, structures not loaded with indicators exhibited THG signals and can also be imaged by the same microscope. They recorded calcium transients at 4 frames/s, with visual stimuli (e.g., sinusoidal gratings) and showed orientation selectivity.

### Simultaneous projection of patterned illumination

The RA microscope discussed earlier facilitates time-shared projection of patterned illumination. However, another way to provide patterned illumination is to divide a single laser into multiple beams and project them in parallel. Alongside time-shared patterned illumination, this technique also found initial applications in optical manipulation of microscopic particles (Dufresne and Grier 1998; Liesener et al. 2000; Eriksen et al. 2002; Curtis et al. 2002; Daria et al. 2003; Rodrigo et al. 2005; Daria et al. 2011). The generalized phase contrast (Glückstad 1996) and holographic projection techniques (Dufresne and Grier 1998) encode phase patterns on a spatial light modulator (SLM) to decompose a single laser into multiple beams. These techniques have now been ported to probe neuronal activity (Nikolenko et al. 2008; Lutz et al. 2008; Papagiakoumou et al. 2010; Dal Maschio et al. 2010; Anselmi et al. 2011; Yang et al. 2011; Go et al. 2012; Go et al. 2013; Ducros et al. 2013; Bovetti and Fellin 2015; Foust et al. 2015; Castanares et al. 2016; Bovetti et al. 2017; Go et al. 2019; Castanares et al. 2019; Castanares et al. 2020).

**Fig. 11:**
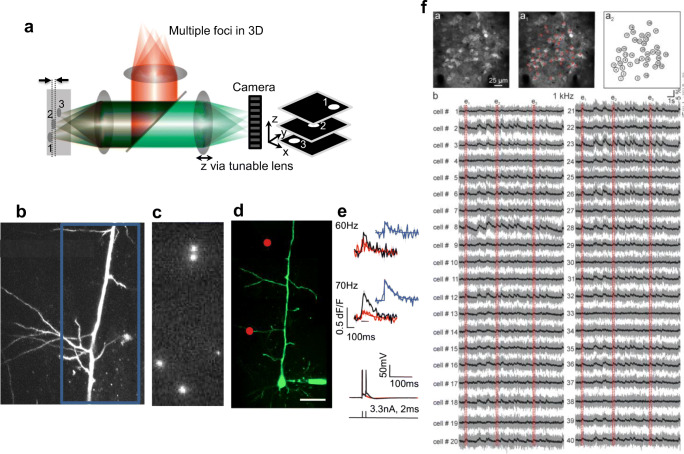
**a** Patterned illumination via a holographic multi-foci 2P microscope. Relative axial discrimination is shown in dashed lines. Inset shows the representative 3D image output. **b** Adapted from Castanares et al. (2020). A flattened *z*-stack image showing the proximal dendritic tree of a L5 pyramidal neuron loaded with Alexa-488 and Cal-520. The scale bar is 50 μm. **c** Holographically projected multiple foci incident on the dendrites sites and fluorescence recorded using an EMCCD camera. **d** An image of L5 pyramidal neuron with holographically projected foci at the oblique branches. **e** Calcium responses during a train of two APs at different frequencies with 70-Hz train evoking a dendritic spike. **f** Adapted from Bovetti et al. (2017). (Top Left) Image via scanning 2P microscope showing the GCaMP6f-expressing neurons. (Top middle and right) The red crosses indicate the positions of the spots used for scanless imaging. (Bottom) Fluorescence signals recorded with the camera during scanless multipoint illumination of the neurons

Holographic projection can project an array of diffraction-limited multiple foci and entails the pre-calculation of a computer-generated hologram (CGH). When the hologram is illuminated, the optical transformation in the far-field (or at the focus of a lens) projects the arbitrary optical field pattern at the sample (see Fig. 11a). In an *in vitro* brain slice preparation, Castanares et al. (2020) studied dendritic spikes by performing scanless imaging or simultaneous holographic illumination of proximal dendritic regions of pyramidal neurons loaded with calcium indicator (Cal520). Figure 11b–e are adapted from Castanares et al. (2020), which show holographic 2P functional calcium imaging at the apical oblique dendrites of a L5 pyramidal neuron. Calcium transients from each holographic site show a stark difference when the neuron is injected with a train of two action potentials at 60 Hz and 70 Hz (Fig. 11e).

Multi-site 2P excitation using a hologram has also been used for scanless functional calcium imaging of Ca^2+^ activity in the intact mouse brain. Bovetti et al. (2017) performed scanless imaging of the activity of pyramidal neurons expressing GCaMP6 in the cortex with an acquisition rate of 1000 frames/s. Figure 11f is adapted from Bovetti et al. (2017), which shows pyramidal neurons about 140-um depth from the dura and calcium transients from 21 pre-selected cortical neurons, imaged via the scanless configuration at 1000 frames/s in an anesthetized mouse.

## Discussion

**Fig. 12:**
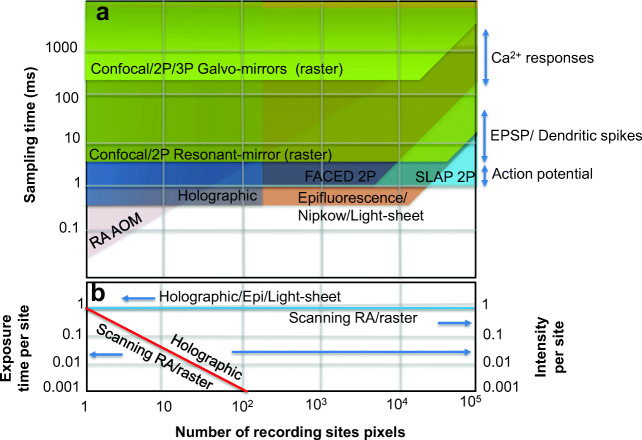
Summary of optical technologies and their spatio-temporal properties

Using light to probe the brain requires imaging technologies capable of meeting the spatio-temporal requirements to analyze the activity of single neurons and neuronal circuits. Figure 12 shows a summary of optical techniques and their spatio-temporal properties. With optical techniques, the minimum spatial resolution is dictated by the diffraction limit and therefore limited to imaging feature sizes of around ~ 1 μm. On the other hand, the spatial range shown in the *x*-axis (in Fig. 12) is the amount of information that can be obtained across the field-of-view, which will depend on the optical magnification and the number of sites/pixels collected as a function of time. The sampling time is shown in the *y*-axis and can be correlated with neuronal events such as the firing of APs, EPSPs, dendritic spikes, and calcium responses (right axis).

An epifluorescence 1P microscope using an EMCCD camera can sample as fast as 0.5 ms (2000 frames/s) depending on the size of the region-of-interest (ROI). Capturing a full-frame results in a longer sampling time. Nonetheless, with limited ROI, a 1P epifluorescence microscope is capable of recording action potentials and dendritic spikes. Confocal microscopes can improve the contrast as it only acquires images within the focal plane. Using galvanometer mirror scanners can provide a sampling time of around 250 ms (4 frames/s) depending on the number of pixels. However, using a resonant scanner to produce a 100 × 1000-pixel image reduces the sampling time to about 5 ms (or 200 frames/s).

High-speed confocal imaging can also be performed via a Nipkow disk, which is highly dependent on the speed of the camera used. Hence, the optimal temporal resolution, for the Nipkow disk, depends on the speed of the camera and the speed of the disk's rotation. Nipkow disk confocal microscopes have been used for recording calcium transients. However, in some cases, it is not fast enough to record voltage spikes. Foust et al. (2010) used the Nipkow disk only for morphological reconstruction of the neurons but used the conventional 1P wide-field imaging for voltage imaging. Another way to improve the contrast in 1P imaging is to restrict the illumination within a single plane. High-speed imaging using light-sheet illumination and the high-speed camera can provide high frame rates and variants of the technique allow for its *in vivo* imaging of neuronal activity in the mouse brain.

With 2P microscopes, RA microscopes are now commonly used for the recording of calcium activity *in vivo*. RA microscopes with AOMs share the sampling time, which means that as the number of recording sites (*N*) is increased, the exposure time per site is reduced by 1/*N* (see Fig. 12b). On the other hand, holographic projection shares the total intensity of the excitation laser into multiple foci. As such, the intensity per site (or per focus) is inversely proportional with *N*. In holographic projection, the system is limited by the total laser power available.

Newly developed high-speed 2P imaging systems have great potential for imaging the electrical activity of neurons *in vivo*. Techniques based on the FACED (Wu et al. 2020) and SLAP (Kazemipour et al. 2019) can provide high-speed imaging with a sampling time of ~ 1 ms (~ 1000 frames/s). With that speed, the electrical activity of neurons expressing GEVIs can be imaged.

Using 3P microscopes allow for imaging deep regions of the intact brain achieving good spatio-temporal resolution (Wang and Xu 2020). With weak 3P fluorescence, scanning speeds for such microscopes will be restricted to using resonant scanning mirrors. Its implementation with holographic projection may be feasible with higher quantum efficiency cameras. While high-speed 3P volumetric calcium imaging has been reported (Rodriguez et al. 2018; Chen et al. 2018), imaging the electrical activity of neurons in an intact brain has not been shown.

## Conclusion

Recent revolutionary developments in photochemical and optogenetic tools have enhanced the link between optics and neuroscience to study the physiology of an individual neuron and its function in networks. The combination of optical recording of calcium and electrical activity of cellular networks requires innovative microscope designs to achieve the required spatio-temporal resolution. Step-by-step, a wide range of techniques have been proposed and tested. Each of them carries significant advantages, but also require tradeoffs. Wide-field 1P imaging using a high-speed camera allows imaging of electrical activity in neurons but is not capable of imaging through optically thick media. On the other hand, multi-photon (2P and 3P) microscopy enables the probing of circuit functions at deeper regions of the brain (Yang and Yuste 2017). These techniques will experience further technical developments in terms of optimized speed and better signal-to-noise ratio.

The current challenge is to improve the spatio-temporal resolution while simultaneously imaging deeper into the brain. Moreover, an even greater challenge is to image through the intact skull which has shown early results (Park et al. 2015; Wang et al. 2018; Yoon et al. 2020) and in the future can provide non-invasive imaging without altering the physiological conditions of the brain. Incorporating wavefront correction schemes can improve imaging through the skull or at deeper regions of the brain (Ji et al. 2010; Park et al. 2015; Choy et al. 2017; Rodriguez and Ji 2018; Yoon et al. 2020).

Optical technologies have certainly delivered new avenues for probing a simple mammalian brain such as those of rodents. While several neuroscience questions on basic brain functions can be answered with rodent brains, more complex brain functions such as intelligence and cognitive ability can only be investigated in more advanced brains such as those in primates. As mentioned earlier, we are just scratching the surface both literally and figuratively. More challenges have yet to come as we aim to understand more complex brain functions that cannot be answered by investigating rodent brains.

These challenges for human creativity will provide us with better solutions, beyond conjectures, theories and Gedanken-experiments, by providing empirical evidence of the detailed operations of neuronal circuits in the brain.
